# Bio-Experiential Technology to Support Persons With Dementia and Care Partners at Home (TEND): Protocol for an Intervention Development Study

**DOI:** 10.2196/52799

**Published:** 2023-12-29

**Authors:** Elizabeth A Rochon, Maimouna Sy, Mirelle Phillips, Erik Anderson, Evan Plys, Christine Ritchie, Ana-Maria Vranceanu

**Affiliations:** 1 Center for Health Outcomes and Interdisciplinary Research Department of Psychiatry Massachusetts General Hospital Boston, MA United States; 2 Mongan Institute Center for Aging and Serious Illness and the Division of Palliative Care and Geriatric Medicine Department of Medicine Massachusetts General Hospital Boston, MA United States; 3 Studio Elsewhere Brooklyn, NY United States; 4 Harvard Medical School Boston, MA United States

**Keywords:** dementia, dyadic, bio-experiential, serious gaming, psychosocial

## Abstract

**Background:**

Alzheimer disease and related dementias are debilitating and incurable diseases. Persons with dementia and their informal caregivers (ie, dyads) experience high rates of emotional distress and negative health outcomes. Several barriers prevent dyads from engaging in psychosocial care including cost, transportation, and a lack of treatments that target later stages of dementia and target the dyad together. Technologically informed treatment and serious gaming have been shown to be feasible and effective among persons living with dementia and their care partners. To increase access, there is a need for technologically informed psychosocial interventions which target the dyad, together in the home.

**Objective:**

This study aims to develop the toolkit for experiential well-being in dementia, a dyadic, “bio-experiential” intervention for persons with dementia and their caregivers. Per our conceptual model, the toolkit for experiential well-being in dementia platform aims to target sustained attention, positive emotions, and active engagement among dyads. In this paper, we outline the protocol and conceptual model for intervention development and partnership with design and development experts.

**Methods:**

We followed the National Institutes of Health (NIH) stage model (stage 1A) and supplemented the model with principles of user-centered design. The first step includes understanding user needs, goals, and strengths. We met this step by engaging in methodology and definition synthesis and conducting focus groups with dementia care providers (N=10) and persons with dementia and caregivers (N=11). Step 2 includes developing and refining the prototype. We will meet this step by engaging dyads in up to 20 iterations of platform β testing workshops. Step 3 includes observing user interactions with the prototype. We will meet this step by releasing the platform for feasibility testing.

**Results:**

Key takeaways from the focus groups include balancing individualization and the dyadic relationship and avoiding confusing stimuli. As of September 2023, we have completed focus groups with providers, persons with dementia, and their caregivers. Additionally, we have conducted 4 iterations of β testing workshops with dyads. Feedback from focus groups informed the β testing workshops; data have not yet been formally analyzed and will be reported in future publications.

**Conclusions:**

Technological interventions, particularly “bio-experiential” technology, can be used in dementia care to support emotional health among persons with a diagnosis and caregivers. Here, we outline a collaborative intervention development process of bio-experiential technology through a research, design, and development partnership. Next, we are planning to test the platform’s feasibility as well as its impact on clinical outcomes and mechanisms of action.

**International Registered Report Identifier (IRRID):**

DERR1-10.2196/52799

## Introduction

### Overview

Approximately 6.7 million Americans have a diagnosis of Alzheimer disease or related dementias (dementia) and rates are continuing to rise with the increasing aging population [1]. Even with the advent of disease-modifying agents [2], dementia remains a debilitating and incurable disease that impacts cognitive, emotional, social, and behavioral functioning [3,4]. In particular, psychological health concerns, such as stress, anxiety, depression, and social isolation, are common across the illness process and impact outcomes among both persons with the diagnosis and their care network.

More than 11 million Americans are the informal caregivers for a person with dementia [5]. Informal caregivers (ie, someone who provides care for a person with dementia not paid through an agency, often a family member or friend) serve a critical role in providing care for persons living with dementia. Caregivers take on a multifaceted role, including providing direct care assistance (ie, activities of daily living), instrumental activities of daily living (eg, finances and shopping), and emotional support. A large body of literature demonstrates that dementia caregivers report high levels of stress, and often, report a greater impact on mental and physical health than informal caregivers of nondementia illnesses and chronic diseases [6,7]. Depression occurs in at least a third of all dementia caregivers [8]. Increased caregiver stress relates to poor caregiver mental and physical health [9,10] and mortality among their care recipients [11].

Psychosocial factors are interdependent between persons with dementia and their caregivers, such that distress from 1 member of the dyad impacts the other. Evidence suggests that psychologically based dyadic interventions may reduce caregiver burden [12-14] and improve quality of life among persons living with dementia [13]. Dyadic interventions that target the person with dementia, the caregiver, and their interpersonal relationship can have synergistic effects on individual interventions and maximally benefit both members of the dyad [15,16].

Several barriers exist to engaging caregivers and persons with dementia in psychosocial interventions. First, many psychosocial interventions require a trained provider and protected time, often around 60-90 minutes a week for 8-12 weeks. Care is costly, and not always covered by insurance, and dyads often face the price of lost wages and transportation [10]. Furthermore, psychosocial care is overly focused on the early stages of dementia, when the focus is often on education and planning [17]. Additionally, there are few interventions that target the dyad together and even fewer in the late stages [18,19].

Technology can help bypass the barriers as interventions can be delivered at home at the convenience of the dyad and without a trained clinician. A growing body of literature supports the use of technology to provide feasible and effective psychosocial interventions among older adults, specifically those living with dementia and their care partners [20]. Technological interventions are available in several modalities for this population, including mobile apps to improve independent living and cognition among persons with dementia [21] or web-based life-review therapy to decrease depression and increase well-being [22].

A complementary area of research suggests that serious gaming, games intended for purposes other than entertainment (ie, learning) [23,24], may be enjoyable and improve mental health outcomes among older adults with dementia [25,26]. Though the field of serious gaming research is dominated by younger adults, evidence suggests that older adults enjoy serious gaming and are motivated by factors including social wellness and mutual participation to engage in serious gaming [27]. Serious gaming engages users in multiple activities and experiences. Similarly, an emerging field that combines elements of serious gaming with multisensory stimulation [28] (ie, “bio-experiential” design) represents an unexplored opportunity to support persons with dementia (including those with advanced symptoms) and their care partners with important benefits for their well-being. Such interventions can provide accessible opportunities for both persons with dementia and their caregivers to reduce stress and depression and improve well-being [29-31].

### Objectives

In this paper, we aim to describe the protocol for the iterative development of the toolkit for experiential well-being in dementia (TEND), a dyadic technology-based intervention for persons with dementia and their caregivers. We partnered with Studio Elsewhere, a design and technology studio to develop the intervention. TEND combines serious gaming with bio-experiential design to provide dyads with the opportunity to engage in choice-based experiences together with the goal of regulating emotions, supporting interpersonal relationships, and increasing well-being. Furthermore, here we describe a model for the codevelopment of interventions through the partnership of design and development experts and interdisciplinary scientists from an idea to a full product.

## Methods

### Overview

**Figure 1:**
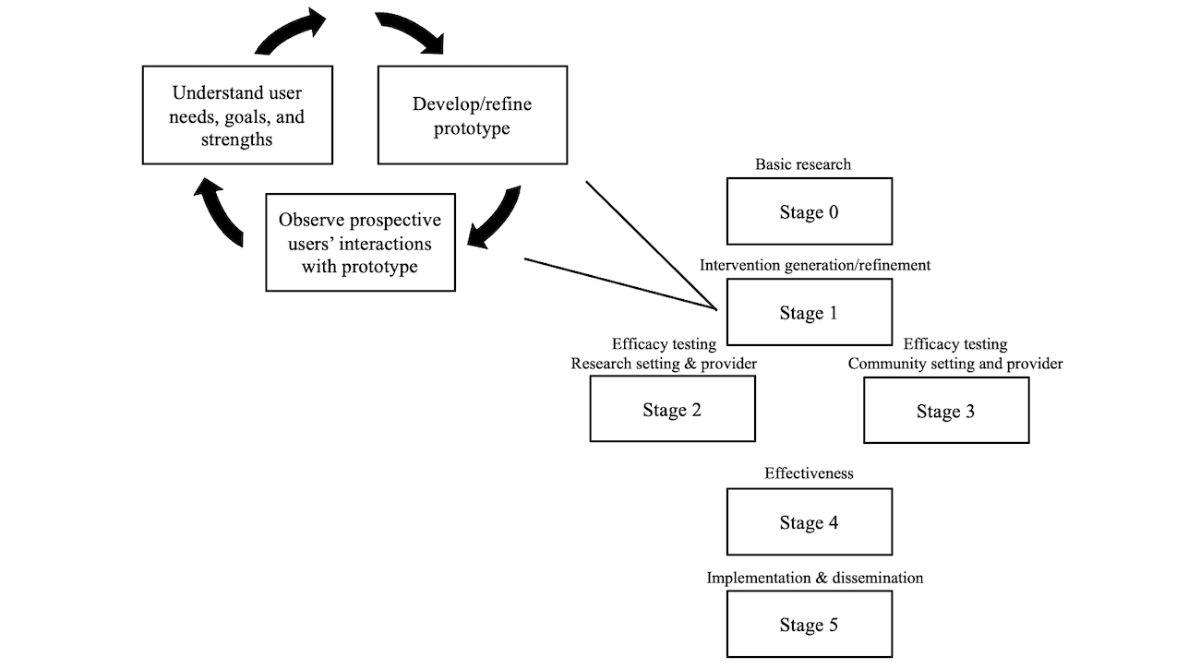
National Institutes of Health stage model 1A: user-centered design.

Our process follows stage 1A of the National Institutes of Health (NIH) stage model for Behavioral Intervention Development [32,33], which includes the generation of our new intervention TEND. NIH stage 1 involves stakeholder feedback, development of the intervention prototype including the conceptual model, testing through an open pilot with an exit interview, followed by refinement of the intervention and conceptual model. Consistent with guidelines for the development of technology-enhanced interventions, we supplement the NIH model with principles of user-centered design as outlined in the process model for user-centered design [34], see Figure 1. This model was appropriate for this study as it allows for intervention development that is highly iterative and places user feedback at the forefront of design choice, which are necessities for promoting the usability of new technology among persons living with dementia [35]. Though steps may be repeated, our process consists of three key steps (and phases): (1) understanding user needs, goals, and strengths, (2) developing and refining the prototype, and (3) observing user interactions with the prototype [36]. These steps align with stage IA of the NIH stage model and position us to progress to feasibility testing (NIH stage IB), after gaining user feedback in this study.

**Figure 2:**
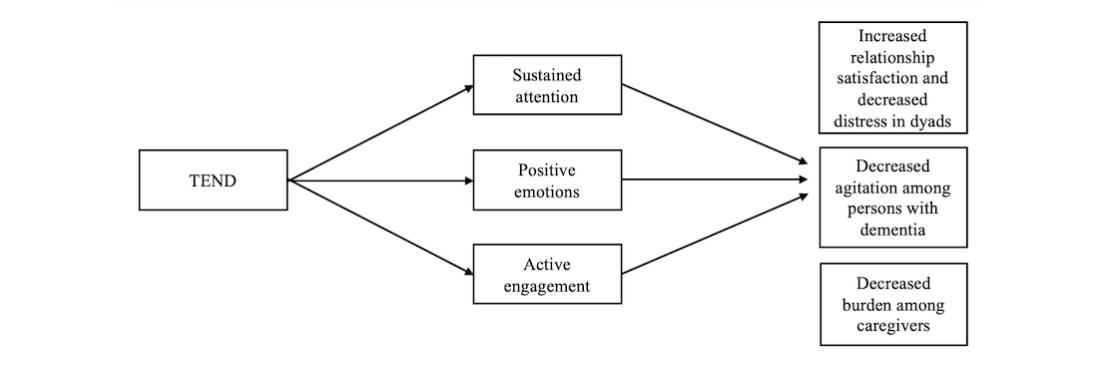
TEND conceptual model. TEND: toolkit for experiential well-being in dementia.

Our initial conceptual model for TEND (Figure 2) is rooted in the theories of flow [37], positive resonance theory [38], and behavioral or unmet needs theory of dementia [39,40]. As the dyad participates together, we expect TEND to engage putative mechanisms of sustained attention, positive emotions, and active engagement, leading to improvement in outcomes of relationship satisfaction, and distress in dyads, agitation among persons living with dementia, and caregiver burden among caregivers [41].

### Ethical Considerations

The Mass General Brigham Institutional Review Board approved all study procedures (2022P001401). Participants reviewed the approved fact sheet and provided verbal consent to all study procedures. In the consent process, participants were informed that all data would be deidentified and that their responses and decision to participate or not participate would not affect the care they received at Mass General Brigham. Providers received US $30 and persons with dementia and their caregivers received US $50 in gift cards respectively for their participation.

### Phase 1: Understand User Needs, Goals, and Strengths

#### Overview

The initial phase represented the discovery process, aimed at establishing the initial parameters and definition of the product. In this phase, we engaged in the following activities: (1) methodology and definition synthesis and (2) key stakeholder focus groups.

#### Methodology and Definition Synthesis

We facilitated communication within our interdisciplinary team by first establishing a common language and definitions. The team members specialize in various disciplines, including clinical psychology, geriatric and palliative medicine, and design technology. As we bridged research expertise with technological and creative design expertise, it was imperative to establish standardized terms. To address possible inconsistencies in terminology, we established a consistently evolving web-based team “dictionary” of agreed-upon definitions. The research, design, and development teams conduct in-person meetings to facilitate definition agreement. Due to the travel burden, weekly web-based meetings are conducted via Zoom (Zoom Video Communications) with the team members, each providing perspectives essential to product development. Team members participate in these weekly meetings to develop a platform by integrating perspectives from engineering, design, research, and clinical medicine together.

#### Key Stakeholder Focus Groups

##### Design

Focus groups were conducted to identify the emotional needs and technology preferences of key stakeholders, including clinicians, persons with dementia, and caregivers. The feedback from persons living with dementia, caregivers, and interdisciplinary dementia care providers was used to guide design choices.

##### Recruitment

We recruited 10 dementia care clinicians from dementia clinics associated with the Departments of Neurology, Psychiatry, and Internal Medicine. We recruited 11 caregivers and persons with dementia from various sources, including the dementia care collaborative program, and through direct contact with Alzheimer disease and other related dementia caregivers within an academic health system. We received institutional review board approval on August 8, 2022. Recruitment flyers were disseminated to referral sources digitally. Interested potential participants were instructed to contact the assigned research assistant. The research assistant confirmed the interest and availability of the potential participants. Inclusion criteria included the age of 18 years or older and self-identified as a dementia care provider, a caregiver for a person living with dementia, or a person living with dementia. After reading the informed consent, the research assistant assessed the persons with dementia’s ability to provide verbal consent through the University of California, San Diego brief assessment of capacity to consent questionnaire [42]. If the person with dementia scored a 1 or 0 on University of California, San Diego brief assessment of capacity to consent questionnaire, suggesting they did not comprehend the purpose of the group interview nor what they would be doing, the research assistant asked them to provide assent and asked their caregiver to provide their verbal consent. Providers and caregivers provided verbal consent to participate.

##### Procedures

Focus groups were conducted over Zoom for approximately 60 minutes. We conducted 5 focus groups separated into groups of dementia care providers, caregivers, and dyads. Users viewed the proposed components of the platform and early content to provide feedback and identify potential uses and gaps. We selected questions for stakeholders based on themes that required high priority early on in the design process. Themes included safety considerations, barriers to use, and utility across subtypes and stages of dementia. As part of the discovery phase, focus groups allowed for generative feedback to steer the direction of the platform toward the confirmed needs and preferences of the users.

##### Analysis

We identified rapid data analysis (RDA) as our analysis plan to facilitate rapid intervention development while maintaining scientific rigor [43-45]. We conducted rapid qualitative analysis because our goal was not to elicit new perspectives or an in-depth understanding of dyadic relationships in dementia but to understand key intervention elements, safety issues, and facilitators and barriers to implementation of the technologically mediated biopsychosocial intervention being developed.

### Phase 2: Developing and Refining the Prototype

#### Overview

Throughout phase II the prototype is refined through a user feedback process to increase program fidelity and user-centeredness [34,36]. Similar to the key stakeholder interviews, we are engaging in a feedback loop to effectively disseminate information from users to the research, design, and development teams. While phase I focused on understanding user needs, goals, and strengths, phase II is characterized by developing and refining the prototype.

#### β Workshops

##### Design

We are implementing up to 20 iterations of platform β testing workshops, a period in which a product (the platform) is released to users to test in a real-world environment to determine any issues prior to initial release [46]. The team presents platform developments to users for iterative collaborative input and feedback on feasibility, satisfaction, and usability.

##### Recruitment

We are recruiting dyads for platform β testing. Similar to focus group recruitment, dyads are recruited from sources including the dementia care collaborative program, and through direct contact with Alzheimer disease and other related dementia caregivers within the internal health system. Inclusion criteria are similar to that of phase I (see above) and include only persons living with dementia and their caregivers. Dyads are invited to participate in multiple workshops, if interested.

##### Procedures

β testing workshops are conducted through Zoom for approximately 90 minutes and are facilitated by research staff and the design and development team. Conditions are intended to replicate a typical user scenario in which persons living with dementia and caregivers use the web-based platform together. Users are presented with a developed platform and toolkit to navigate. Research, design, and development staff are able to directly observe the usability of the platform. Furthermore, design and development staff inquire about platform use and acceptability to amend features and guide the next iteration of β testing.

##### Data Collection

Research staff elicit qualitative data from participants through exit interview questions gathering feedback on the platform. Additionally, staff interview participants with a series of quantitative questionnaires, including an adapted feasibility scale [47] and an adapted system usability scale [48]. Research staff records the interview to engage in behavioral observation using the Video Coding–Incorporating Observed Emotion scale [49,50] to gauge emotion, verbal engagement, visual engagement, collective engagement, and agitation among persons with dementia.

##### Data Analysis

We will conduct RDA. The analysis plan is similar to that of phase I (see above). We will review the recorded interviews to examine changes in emotion, engagement, and agitation among persons with dementia and the correlating platform features.

## Results

As of August 2023, we have completed phase I. Focus group qualitative data has not yet been formally analyzed outside of RDA and will be reported in future publications. Key takeaways from the focus groups included prioritizing individualization, avoiding potentially confusing stimuli, and focusing on the dyadic relationship. Findings from the focus groups informed the β testing workshops. As of September 2023, we have completed 4 iterations of platform β testing workshops.

## Discussion

### Principal Findings

Guided by the process model for user-centered design [34] and the NIH stage model [32], above, we have delineated the initial process of user-centered design for a partnership between design and development experts and interdisciplinary scientists. We have engaged in codevelopment through methodology and definition synthesis, focus groups, and β workshops (currently ongoing). Combining serious gaming with bio-experiential design to develop the initial TEND platform, we aim to regulate emotions, support interpersonal relationships, and increase well-being. As bio-experiential technology emerges as a promising field within health care, we have the opportunity to mitigate traditional barriers to effective and equitable dementia care.

Within the development of this bio-experiential design, we outline an effective strategy for academic and design and development team partnership. Multidisciplinary team partnerships are especially beneficial in developing technologies for dementia, as scientific findings and procedures may support adaptations and user experiences [51]. For example, by partnering with researchers, developers are able to address cognitive and functional deficits through adaptations to technology including customization, errorless learning [52], and simple narratives [53]. We adapted to the challenges of codevelopment between distinct teams by establishing a common language and definitions and weekly meetings. The web-based, immersive platform and toolkit developed by Studio Elsewhere is guided by a concept generation and feedback loop incorporating key stakeholders throughout phases I and II.

Through aims 1 and 2 of our partnership, we expect to develop the experimental platform and refine and test the platform. We anticipate these efforts will result in a free interactive, immersive bio-experiential web platform and accompanying toolkit, after subsequent testing for efficacy in a large group of dyads. We anticipate that the resulting technologies will be easily implemented in the home to engage persons with dementia and caregivers in meaningful and relationship-enhancing activities. Consistent with existing literature on technology adaptations for older adults and persons living with disabilities, this study demonstrates various user design considerations, including cognitive, communication, sensory, and physical deficits common with dementia and advancing age [54,55]. In particular, our team is tasked with designing technology that is engaging for both persons with dementia and caregivers, who likely present with varying cognitive abilities and technological literacy. Further, the technology must be appealing and usable across dyads. Our approach outlined here will result in a bio-experiential technology that will be ready for more rigorous testing in the next phase of the work. Following these phases, we aim to conclude with the final stages of user-centered design, observing prospective users’ interactions with the prototype [34]. In future studies, we will optimize the platform through engaging in pilot testing and efficacy testing with the goal of wide implementation.

As the field of technological interventions and serious gaming expands and the need for cost-effective and dyadic interventions increases, there is an urgency for technology-enhanced dyadic and psychosocial intervention in dementia informed by behavioral science. Not only do persons with dementia and caregivers find psychological benefit from technological interventions, but they are often highly enjoyable. Here, we outline a highly collaborative intervention development model to address traditional barriers and promote psychosocial wellness among this population through a bio-experiential intervention.

### Limitations

We anticipate several limitations with this research. Participants must have access to technology equipped with audio and video capture devices to participate in focus groups and workshops, which may exclude low socioeconomic status participants. Additionally, while we engage in β testing workshops over Zoom to replicate real-life environments, the presence of research personnel and design and development team members may unintentionally influence participant reporting on feasibility and usability scales. Furthermore, in research with persons with advanced dementia, the reliability of data is a concern for participants and proxies.

### Conclusions

In this protocol, we outline the development of bio-experiential technology for persons with dementia and their caregivers, as well as the partnership between design and development experts and interdisciplinary scientists. This program of research has the potential to address clinical challenges (eg, depression and agitation) among persons with moderate-severe dementia, a population with few evidence-based treatments, and improve outcomes among dyads. Further, the ability to deliver a technology-based intervention in the home has implications for increasing access and translatability. The novelty of a cognitively mismatched dyadic intervention developed based on user-centered design principles will provide a methodological and conceptual framework for future investigators in this field.

## Appendix

Multimedia Appendix 1 Peer-reviewer report from Robert Wood Johnson Foundation.

## Abbreviations

NIH: National Institute of Health
RDA: rapid data analysis
TEND: toolkit for experiential well-being in dementia

## Data Availability

The data sets generated and analyzed during this study are available from the corresponding author on reasonable request once the trial is complete.
