# Supplementary material for: Bio-Experiential Technology to Support Persons With Dementia and Care Partners at Home (TEND): Protocol for an Intervention Development Study
Source: JMIR Res Protoc. 2023 Dec 29;12:e52799. doi: 10.2196/52799 (PMC10787328; doi:10.2196/52799)
Supplement: Multimedia Appendix 1 [file resprot_v12i1e52799_app1.pdf]

50 College Road East
Princeton, NJ 08540-6614
Tel. 877 843 RWJF (7953)
www.rwjf.org

Robert Wood Johnson Foundation

October 12, 2023

Dr. Christine Ritchie
Director, Mongan Institute Center for Aging and Serious Illness
Massachusetts General Hospital

Dear Dr. Chirstine Ritchie:

Below are the questions and comments that came out of the review process. Thank you for your responses, also captured below.

Warm regards,

Devin Woods
Program Officer, Pioneering Ideas for an Equitable Future

Focus Groups

The number of co-design sessions and participants seems low. Could this number be increased? Suggestions for this included (1) increased numbers of participants and focus groups and (2) multiple interactive and iterative design sessions with each target group throughout the course of the design process and perhaps the project.

RESPONSE: We will be conducting a minimum of 4 longitudinal focus groups (e.g., series of focus groups with the same participants over time across 3 phases: 1) Discovery, 2) development and 3) feedback from implementation. We will also use multiple individual feedback sessions with participants. The combination of focus groups and individual feedback sessions will allow for multiple interactive and iterative opportunities throughout the course of the design process. A recent systematic review of qualitative studies shows that 4 focus groups are generally sufficient to gather nuanced information from participants; however, we are prepared to increase the numbers of focus groups and participants if new, rich information continues to be gleaned from each focus group. Our team (Ritchie and Vranceanu) have extensive expertise with qualitative methods and focus groups.
Reference: Hennink M, Kaiser BN. Sample sizes for saturation in qualitative research: A systematic review of empirical tests. Social Science & Medicine, 2001 Nov 2.
free access at: https://pubmed.ncbi.nlm.nih.gov/34785096/

Can you provide greater detail on how the focus groups will be conducted? Will they be online, in person, or up to the preference of the participants?

RESPONSE: Focus groups will include up to 10 individuals. We will use a semi-structured qualitative script. Groups will be facilitated by a trained clinical psychologist with dementia expertise, trained by the Co-PIs. Focus groups will aim to gather feedback on the proposed bio-experiential platform content and procedures. Participants will respond to prompts about: 1) the proposed content of the platform 2) barriers and facilitators to participation and 3) case-based scenarios for tailoring platform content. Participants will be informed that there is no right or wrong answer. The facilitator will encourage an open discussion. Each focus group will last 60-90 minutes and will be delivered virtually using a secure Zoom platform. Groups will be audio recorded and information will be transcribed and analyzed to inform the bio-experiential platform content, and study procedures.

However the focus groups are conducted, this will impact who can participate. Can you share how this is being considered in the design of the project?

RESPONSE: We decided to conduct the focus groups virtually for 3 main reasons. First, our larger goal is to develop a platform that meets the needs of geographically diverse people with dementia and their caregivers. Virtual focus groups allow us to use our dementia national organization partners and thus increase representation of our sample. Second, we want to meet our proposed timeline and not be slowed down by the current pandemic including the recent Omicron variant, which now poses heightened risk for older individuals and prevents safe in person gatherings for focus groups. Our team has extensive experience in conducting both in person and virtual focus groups, and the quality of the data collected is similar. Third, our end product will be a virtual program. Conducting virtual focus groups will allow us to begin to understand our participants' challenges (e.g., hearing impairment, digital literacy) so that we can start addressing them through our iterative prototyping as early as possible in the design process.

Project and Impact

Can you share more about how you envision caregivers and patients will have access to the equipment, tools, and knowledge to ensure the web platform is usable for them?

RESPONSE: The only equipment that the patients and caregivers need is a tablet, computer, laptop or smartphone to participate in the focus groups and have access to the web platform. As part of this project we will develop a toolkit that will be available to all participants. No knowledge would be required in order to participate. Studio Elsewhere has developed a digital literacy lab and therefore has experience with guiding technology naive participants in the work. Our team also has experience with developing protocols that effectively engages older adults in the use of technology (and is currently part of a NIH-funded grant). Our qualitative work in the focus groups will address ensuring that the web platform is usable for the participants.

For this project, we will rely on our participants having access to a tablet, laptop, desktop computer or smartphone. Most US families have access to at least one computer, laptop, smartphone or tablet, though we acknowledge that some might not be able to participate in this study due to lack of technology. In future work, we will plan to provide tablets; with proposed resources, this is not feasible.

Can you share more detail on what the role of the clinicians will be in a tool that is targeted to patients and caregivers?

RESPONSE: We will enroll clinicians who have expertise working clinically with persons with dementia and their caregivers. Thus, they are intimately aware of multiple challenges faced by these individuals, and will thus provide important information and insights over and above that provided by patients and caregivers themselves. Clinician feedback will thus enrich the qualitative information collected so that we can develop a tool that meaningfully supports participants. In future projects, we hope to integrate the web platform we will develop as part of clinical care, to supplement medical visits. By gathering information from clinicians we can more efficiently move toward scalability and dissemination. Studio Elsewhere has used a similar approach in prior projects.

The proposal identifies a number of stressors (i.e. navigating medical system, managing challenging behaviors in the care recipient, concerns about the quality of their caregiving and

impact of caregiving on their own health, and social isolation) as well as citing racism and discrimination as barriers, can you provide more detail on how you envision the web platform will help alleviate these stressors?

RESPONSE: The goal of this web-based platform is to give persons living with dementia and caregivers access to a tool that helps them mitigate the stress response which is triggered by any events that are perceived as stressful by the individual. The platform will help downregulate participants' perceived stress regardless of its source (e.g., racism, social isolation, navigating medical systems).

Considering that the selection of community partners and participants has not begun, the time frame for this project seems to be very quick and given that identifying participants may take some time, is the current time frame realistic?

RESPONSE: Drs. Ritchie and Vranceanu have long standing partnerships with local and national caregiver organizations, in addition to MGH. We will recruit potential participants from 3 main sources: 1) the Dementia Collaborative Care (DCC) program at MGH (which Dr. Ritchie directs and receives referrals from MGH Neurology, Geriatrics and the larger Boston area); 2) Massachusetts Alzheimer's Disease Research Center (Dr. Ritchie co-directs the outreach and recruitment core); and 3) nationally from several sources including the National Alliance of Caregiving (Dr. Vranceanu has a longstanding relationship with them). Our community partners are aware of this project (they submitted letters of support) including our timeline, and are excited to work with us on this project.
